# Differential evolution of diabetic ketoacidosis in adults with pre-existent versus newly diagnosed type 1 and type 2 diabetes mellitus

**DOI:** 10.1186/s12902-023-01446-8

**Published:** 2023-09-12

**Authors:** Fateen Ata, Adeel Ahmad Khan, Ibrahim Khamees, Baian Z. M. Mohammed, Haidar Hussein Barjas, Bassam Muthanna, Mohammed Bashir, Anand Kartha

**Affiliations:** 1 Department of Endocrinology, Hamad General Hospital, Hamad Medical Corporation, 3050 Doha, Qatar; 2 Department of Internal Medicine, University of Missouri-Kansas City, Kansas City, MO USA; 3 Department of Internal Medicine, Hamad Medical Corporation, Doha, Qatar; 4 Department of Geriatrics, University of Illinois College of Medicine, Chicago, USA; 5 Qatar Metabolic Institute, Doha, Qatar; 6 Weill Cornel Medicine, Doha, Qatar

**Keywords:** Diabetic ketoacidosis, DKA, Type 1 Diabetes, Type 2 Diabetes

## Abstract

**Background:**

Diabetic ketoacidosis (DKA) was once known to be specific to type-1 diabetes-mellitus (T1D); however, many cases are now seen in patients with type-2 diabetes-mellitus (T2D). Little is known about how this etiology shift affects DKA's outcomes.

**Methods:**

We studied consecutive index DKA admissions from January 2015 to March 2021. Descriptive analyses were performed based on pre-existing T1D and T2D (PT1D and PT2D, respectively) and newly diagnosed T1D and T2D (NT1D and NT2D, respectively).

**Results:**

Of the 922 patients, 480 (52%) had T1D, of which 69% had PT1D and 31% NT1D, whereas 442 (48%) had T2D, of which 60% had PT2D and 40% NT2D. The mean age was highest in PT2D (47.6 ± 13.1 years) and lowest in PT1D (27.3 ± 0.5 years) (*P* < 0.001). Patients in all groups were predominantly male except in the PT1D group (55% females) (*P* < 0.001). Most patients were Arabic (76% in PT1D, 51.4% in NT1D, 46.6% in PT2D) except for NT2D, which mainly comprised Asians (53%) (*P* < 0.001). Patients with NT2D had the longest hospital length of stay (LOS) (6.8 ± 11.3 days) (*P* < 0.001), longest DKA duration (26.6 ± 21.1 h) (*P* < 0.001), and more intensive-care unit (ICU) admissions (31.2%) (*P* < 0.001). Patients with PT1D had the shortest LOS (2.5 ± 3.5 days) (*P* < 0.001), DKA duration (18.9 ± 4.2 h) (*P* < 0.001), and lowest ICU admissions (16.6%) (*P* < 0.001).

**Conclusions/interpretation:**

We presented the largest regional data on differences in DKA based on the type and duration of diabetes- mellitus (DM), showing that T2D is becoming an increasing cause of DKA, with worse clinical outcomes (especially newly diagnosed T2D) compared to T1D.

## Introduction

Diabetic ketoacidosis (DKA) is one of the most common acute complications of diabetes mellitus (DM). The mortality rates secondary to DKA have significantly improved over time (from around 15% in the 1980s to < 1% in 2020) [1, 2]. However, it remains a common cause of mortality in patients with DM, especially type 1 DM (T1D), with up to 76% of deaths related to DKA [3]. The prevalence of DKA in patients with T1D is variable, ranging from 10 to 128 episodes per 1000 people per year, with higher rates reported in poorly controlled DM [3]. Patients with type 2 DM (T2D) are insulin-resistant rather than deficient, which is why DKA is less commonly seen in this population [4]. However, insulin resistance results in the inability of the body to fully respond to the glucogenic and lipolytic effects of insulin, leading to a relative insulin deficiency [4]. The inadequacy of insulin function is further potentiated in the presence of certain precipitating factors. Illness or infections can precipitate DKA in patients with T2D via a surge of counter-regulatory hormones (such as glucagon, cortisol, and growth hormone), rendering the body unable to adequately control lipolysis [4]. Hyperglycaemia in patients with T2D also amplifies the effects of these hormones, making the body prone to DKA [5]. The hyperglycaemic state also reduces insulin response at the molecular level by curbing the transcription of insulin gene [4]. Additionally, insulin deficiency is not uncommon in progressed T2D and can lead to DKA even without a precipitating factor [4].

The true prevalence of DKA in T2D remains unknown. However, an increasing number of DKA cases are seen in T2D [6]. There are multiple potential factors behind the increasing incidence of DKA. First, more patients are now diagnosed with DM [7, 8]. This increasing prevalence of DM, which indirectly increases the occurrence of DKA, may be secondary to diagnosis at a younger age which is associated with worse outcomes in both T1D and T2D [9]. Rising risk factors such as obesity, sedentary lifestyles, and poor dietary habits, among other factors, are also responsible for the increasing prevalence of T2D [6]. Additionally, DKA affects the younger population who are not routinely screened for DM-2 and go unrecognized. Hence, DKA becomes the presenting symptom in this undiagnosed population, usually secondary to a precipitating factor [10]. A third reason is that the younger onset of Diabetes is associated with rapid progression in beta-cell failure and insulin deficiency, making those patients DKA-prone when faced with any precipitating factor such as infections [11].

Amid a rising occurrence of DKA admissions among both T1D and T2D, little is known about the effects of both the duration and the type of DM on the clinical course and outcomes of patients with DKA. Newton et al. reported the differences in the DKA population based on duration and type of DM in 176 patients. Although these groups differed in demographics and biochemical results, the authors did not report DKA outcomes in these groups. One notable factor could be the small number of patients in some groups (5 patients in the newly diagnosed T2D group) [12]. In a 15-year retrospective nationwide analysis, Zhong et al. reported a steady rate of DKA occurrence in patients with T1D in the initial years, with a subsequent rising trend. In contrast, a constant rise was seen in DKA cases among patients with T2D over the years [13]. They reported a lower hospital length of stay and reduced readmission rates in patients with T1D. In a recent cross-sectional analysis of 99 patients, Charoenpiriya et al. reported no difference in the DKA-related outcomes in patients with either type of DM apart from a lower duration of DKA in T2D compared to T1D [14]. Considering variable results of the effects of the type of DM and scarcity of data regarding the effects of duration of DM on DKA-related outcomes, it is critical to review patients admitted with DKA, analysing the outcomes based on type and duration of DM, to identify the population most at risk of adverse outcomes.

In this study, we aimed to review all patients with index DKA admissions to observe the demographic and clinical differences, as well as differences in the outcomes based on the type and duration of DM among patients presenting with DKA.

## Research design and methods

### Study design

We retrospectively reviewed all consecutive patients with index DKA admissions from January 2015 to March 2021 in four general hospitals of Hamad Medical Corporation, Qatar.

### Patient selection

Patients were included according to the following criteria: adult patients (14 years and above) with index DKA admissions within the study period. Patients either had a known DM diagnosis or were newly diagnosed with DM on admission. Hyperglycemia was not used to identify DKA to include euglycemic DKA (EDKA) patients.

Patients who did not fulfil the American diabetes association (ADA) DKA criteria were excluded. Patients with ketoacidosis secondary to other conditions (starvation ketoacidosis, alcohol ketoacidosis) were also excluded. Additionally, recurrent DKA admissions from the same patient were excluded and not analysed. Patients were categorised into T1D and T2D and were further subdivided into new or pre-existing DM. We used International Classification of Diseases (ICD) codes to identify T1D and T2D in the EMR. We validated the accuracy of these codes in a subgroup of patients by looking at C-peptides and Anti-GAD antibodies where available. However, the levels were unavailable in most patients, so validation could not be done for all patients. Diagnosis of the type of DM relied mainly on the clinical judgment of the treating physicians.

### Data collection

Data were extracted from the electronic health record, Cerner Millennium. Collected data included patients' demographic details, laboratory investigations on admission, and outcomes. We collected serum glucose, bicarbonate, lactate and PH levels in the following intervals: first value at admission, within the next 2 h (± 30 min), 4 h (± 30 min), and 6 h (± 30 min).

BMI was categorised into normal, overweight, and obese according to ethnicity. In Asians BMI was categorised as follows; normal < 23 kg/m^2^, overweight 23–24.9 kg/m^2^, obese ≥ 25 kg/m^2^. In all other ethnicities, BMI was defined as follows; normal < 25 kg/m^2^, overweight 25–26.9 kg/m^2^, obese ≥ 27 kg/m^2^. Patients were labelled as having high metabolic risk if one or more factors, including obesity, hypertension, and dyslipidemia, were identified.

DM was categorised as Newly diagnosed versus pre-existing T1D and T2D. NT1D group was defined as patients with DKA who were not known to have DM prior to admission and were subsequently diagnosed as having DM with positive anti-glutamic acid decarboxylase (GAD) antibodies (Ab) and/or low c-peptide levels. NT2D group was defined as patients with DKA who were not known to have DM prior to admission and were subsequently diagnosed as having DM with negative anti-GAD Ab and/or detectable c-peptide levels. Similarly, the PT1D group was defined as patients with T1D diagnosed before index admission with positive anti-glutamic acid decarboxylase (GAD) antibodies (Ab) and/or low c-peptide levels. Lastly, the PT2D group was defined as patients with T2D diagnosed before index admission with negative anti-glutamic acid decarboxylase (GAD) antibodies (Ab) and/or detectable c-peptide levels.

### Outcomes

The outcomes studied among the cohort of patients with index DKA admissions included the point prevalence of the type of DM, DKA severity, and serial biochemical evolution of DKA (serial PH, bicarbonate, lactate, and random blood glucose levels) in both DM types. DKA was diagnosed and categorised into mild, moderate or severe per the ADA DKA diagnostic criteria (using PH, bicarbonate, and anion gap to categorise the severity) [15]. Random blood glucose was not used to diagnose or categorise DKA, so euglycemic DKA cases could be included. Other outcomes included differences in the ICU admission, duration of DKA (hours), the hospital LOS the hospital (days), and in-hospital mortality among the four groups (NT1D, PT1D, NT2D, and PT2D).

### Statistical analyses

We used Stata version 15 for the statistical analysis. We summarised continuous variables as means (standard deviation (SD)) or medians (interquartile range (IQR)) as appropriate and categorical variables as percentages. Continuous variables were compared using the one-way ANOVA ("analysis of variance"), while Chi-square and Fisher's exact test was used to compare categorical variables. We considered a *p*-value ≤ 0.05 as significant.

## Results

### Demographics

Of the 922 patients admitted with index DKA episodes during the study period, 480 patients (52%) had T1D, and 442 (48%) had T2D. The median duration of DM (at the time of admission) in patients with T1D and T2D was 7 (IQR 4–12) years and 5 (IQR 3–12) years, respectively (*P* value < 0.001). The mean ages were 27.3 ± 0.5 years in pre-existing T1D (PT1D), 26.5 ± 9.6 years in new T1D (NT1D), 47.6 ± 13.1 years in pre-existing T2D (PT2D) and 42.6 ± 10.7 years in new T2D (NT2D) (*P* value < 0.001). PT1D were predominantly females (55%), while the other three groups were predominantly males (72.3% in NT1D, 68.4% in PT2D, and 77.3% in NT2D (*P* value < 0.001). Comorbidities, including hypertension, dyslipidemia, and DM micro and macrovascular complications, were mainly found in patients with PT2D. Among the patients with NT2D, hypertension was present in 51 (28.9%), dyslipidemia in 22 (12.5%), microvascular complications of DM in 10 (5.6%), and macrovascular complications in 9 (5.1%) patients (*P* value < 0.001). High metabolic risk was seen in 62.4% of patients with PT2D and 61.9% with NT2D. In comparison, a high metabolic risk was present in 25.6% of patients with PT1D and 22.9% of patients with NT1D (*P* value < 0.001). Diabetes educator visits ranged from 56.8% in NT2D to 18% in PT1D (*P* value < 0.001). Detailed patients' demographics are presented in Table 1. Medication history for DM management was extracted from the EMR for pre-existing DM. Among the 332 patients with PT1D, basal insulin, bolus insulin and premixed insulins were prescribed to 155 (46.7%), 161 (48.5%), and 43 (12.9%) patients, respectively. Many patients were taking insulins from private facilities, data for which was not extractable from the EMR. Among the 266 patients with PT2D, basal insulin, bolus insulin and premixed insulins were prescribed to 77 (30%), 61 (23%), and 33 (12.4%), respectively. Sodium-glucose co-transporter 2 inhibitors (SGLT2i) were prescribed to 33 (12.4%), sulfonylureas to 29 (11%), metformin to 126 (47.4%) patients, thiazolidinediones to 8 (3%) patients, Glucagon-like peptide 1 agonists (GLP-1A) to 8 (3%) patients, and dipeptidyl peptidase-4 inhibitors (DPP-i) to 57 (21.4%) patients.

**Table 1:** Baseline characteristics of new and pre-existing type 1 and type 2 diabetes mellitus patients with index DKA admissions. Data presented as Median (IQR), Mean (± SD), and N (%) as appropriate

Baseline characteristics	T1D (480)		T2D (442)		Significance (*P* value)
	**PT1D** **(332)**	**NT1D** **(148)**	**PT2D** **(266)**	**NT2D** **(176)**	
**Age (years)** (**mean ± sd)**	27.3 ± 0.5	26.5 ± 9.6	47.6 ± 13.1	42.6 ± 10.7	< 0.001
**Gender**					< 0.001
Male	150 (45.2)	107 (72.3)	182 (68.4)	136 (77.3)	
Female	182 (54.8)	41 (27.7)	84 (31.6)	40 (22.7)	
**Ethnicities**					< 0.001
Arabic	252 (76)	76 (51.4)	124 (46.6)	50 (28.4)	
Asian	46 (13.9)	42 (28.4)	119 (44.8)	93 (52.8)	
Africans	23 (6.9)	26 (17.6)	15 (5.6)	26 (14.8)	
Others	11 (3.3)	4 (2.7)	8 (3)	7 (4)	
**BMI** kg/m^2a^					< 0.001
**Asians**					
Normal (< 23)	35 (76.1)	24 (58.5)	45 (38.5)	33 (35.9)	
Overweight (23–24.9)	8 (17.4)	3 (7.3)	21 (17.9)	12 (13)	
Obese (≥ 25)	3 (6.5)	14 (34.1)	51 (43.6)	47 (51.1)	
**Others**					< 0.001
Normal (< 25)	189 (66.5)	70 (67.3)	59 (40.1)	20 (24.1)	
Overweight (25–26.9)	54 (19)	22 (21.1)	41 (27.9)	33 (39.7)	
Obese (≥ 27)	41 (14.4)	12 (11.5)	47 (31.9)	30 (36.1)	
**Comorbidities**					
Dyslipidaemia (128)	30 (9)	6 (4)	70 (26.3)	22 (12.5)	< 0.001
Hypertension (199)	40 (12)	5 (3.3)	103 (38.7)	51 (28.9)	< 0.001
Stroke (260)	3 (0.9)	0	16 (6)	7 (3.9)	< 0.001
Coronary artery disease (56)	13 (3.9)	2 (1.3)	32 (12)	9 (5.1)	< 0.001
Retinopathy (78)	33 (9.9)	0	39 (14.6)	6 (3.4)	< 0.001
Nephropathy (60)	19 (5.7)	0	35 (13.1)	6 (3.4)	< 0.001
Diabetic Foot (25)	5 (1.5)	0	18 (6.7)	2 (1.1)	< 0.001
Microvascular complications of DM (95)	36 (10.8)	0	49 (18.4)	10 (5.6)	< 0.001
Macrovascular complications of DM (56)	13 (3.9)	2 (1.3)	32 (12)	9 (5.1)	< 0.001
Metabolic risk (394)	85 (25.6)	34 (22.9)	166 (62.4)	109 (61.9)	< 0.001
**Consult to diabetes educator (348)**	60 (18)	68 (45.9)	120 (45.1)	100 (56.8)	< 0.001

*Abbreviations*: *BMI* body mass index, *DKA* diabetic ketoacidosis, *DM* diabetes mellitus, *n* number of patients, *sd* standard deviation, *PVD* peripheral vascular disease, *T1D* type 1 diabetes mellitus, *T2D* type 2 diabetes mellitus, *PT1D* pre-existing type 1 diabetes mellitus, *NT1D* new type 1 diabetes mellitus, *PT2D* pre-existing type 2 diabetes mellitus, *NT2D* new type 2 diabetes mellitus

^a^Missing data < 1%

### Laboratory investigations

**Fig. 1:**
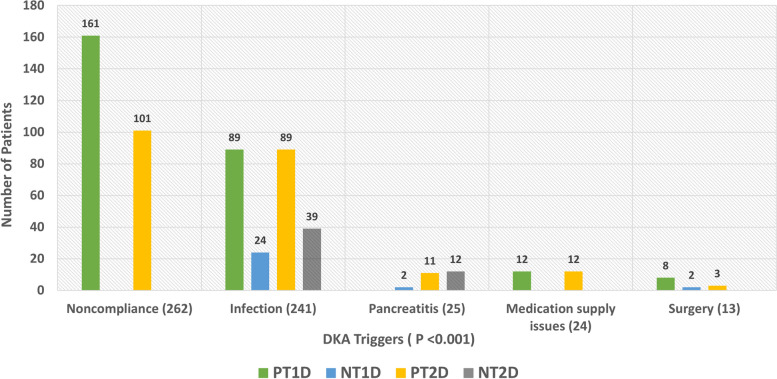
Triggering factors in patients with NT1D (*n* = 148), PT1D (*n* = 332), NT2D (*n* = 176) and PT2D (*n* = 266) with index DKA admissions

**Fig. 2:**
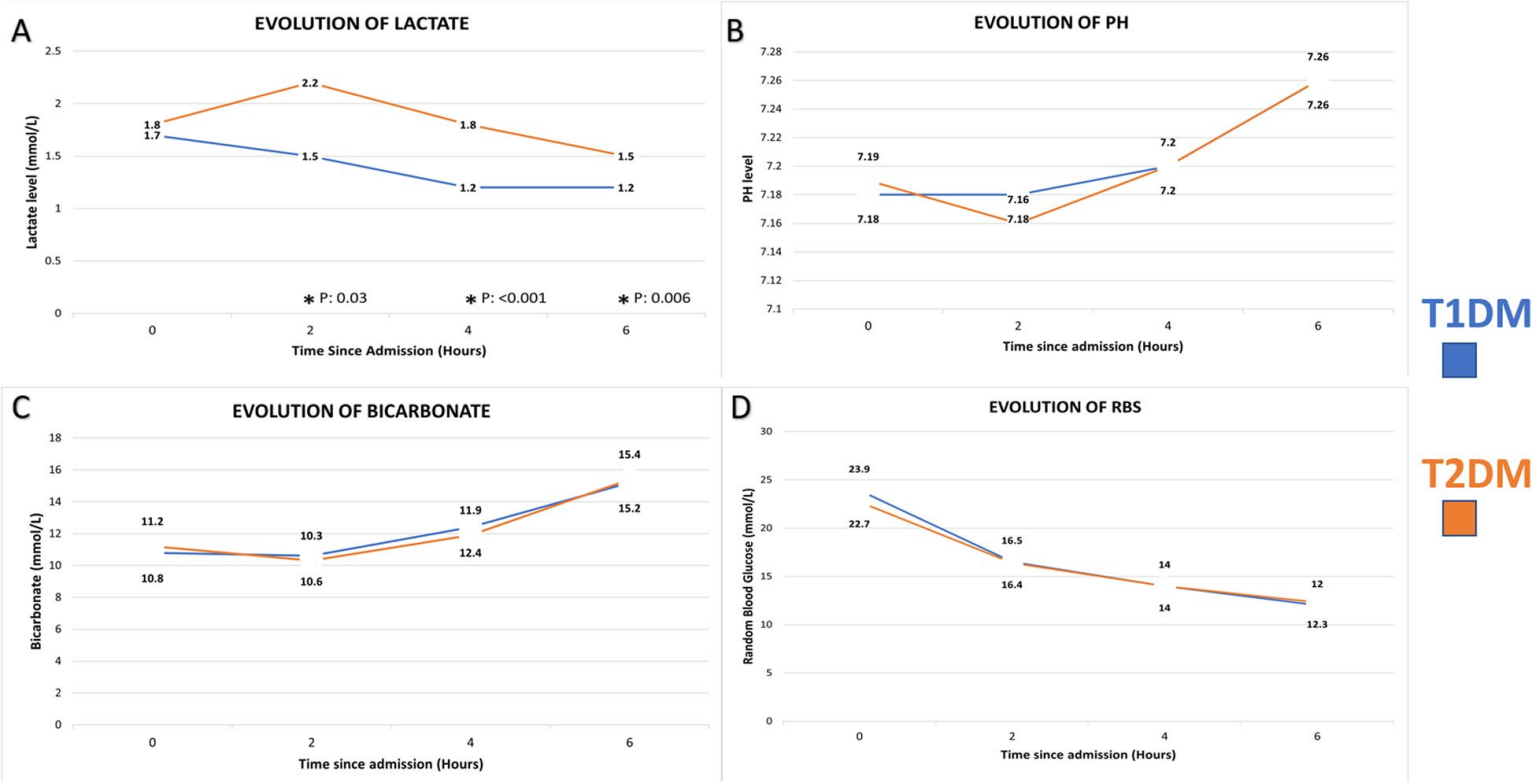
The sequential evolution (values at admission, 2, 4, and 6 h after initiation of DKA management) of DKA in patients* with T1D and T2D. **A** (serial lactate), **B** (serial PH), **C** (serial bicarbonate levels), **D** (serial random blood glucose levels). *In patients with T1D: Lactate at admission (*n* = 318), 2 h (*n* = 192), 4 h (*n* = 200), 6 h (*n* = 217). Serum PH at admission (*n* = 480), 2 h (*n* = 190), 4 h (*n* = 263), 6 h (*n* = 251). Serum bicarbonate at admission (*n* = 480), 2 h (*n* = 251), 4 h (*n* = 285), 6 h (*n* = 312). Random glucose level at admission (*n* = 480), 2 h (*n* = 385), 4 h (*n* = 398), 6 h (*n* = 417). *In patients with T2D: Lactate at admission (*n* = 337), 2 h (*n* = 231), 4 h (*n* = 246), 6 h (*n* = 266). Serum PH at admission (*n* = 442), 2 h (*n* = 225), 4 h (*n* = 290), 6 h (*n* = 274). Serum bicarbonate at admission (*n* = 442), 2 h (*n* = 281), 4 h (*n* = 296), 6 h (*n* = 341). Random glucose level at admission (*n* = 442), 2 h (*n* = 371), 4 h (*n* = 386), 6 h (*n* = 404)

Table 2 summarises the laboratory investigations at admission. The mean glycated haemoglobin (HBA1C) values in patients were: 11.1% ± 2.5 in PT1D, 12.9% ± 2.6 in NT1D, 11.9% ± 2.9 in PT2D, and 12.5% ± 2.8 in NT2D (*P* value 0.3). Anti GAD abs result at the time of index DKA admission were documented for 138 patients in total. Of the 51 patients with NT1D who had Anti GAD Abs documented, 30 (58.8%) had positive titres. Similarly, among 41 patients with NT2D with documented Anti GAD Abs, 3 (7.3%) had positive titres. Twenty-nine patients with PT2D had Anti GAD Abs checked, 6 (20.7%) having positive titres. Lastly, 17 patients with PT1D had Anti GAD abs checked, with 9 (52.9%) being positive. The creatinine clearance ranged from 96.6 ± 96.8 ml/min in patients with PT2D to 103 ± 51.5 ml/min in NT1D (*P* value < 0.001). The C-reactive protein (CRP) varied from 86.8 ± 136.5 mg/L in patients with NT2D to 26.2 ± 56.5 mg/L in patients with NT1D (*P* value < 0.001). Various triggering factors were reported, with non-compliance and infection being the most common (overall and in each group). Non-compliance was reported as a triggering factor in 33.5% of patients with T1D and 23% of patients with T2D. Infection was the precipitating factor of DKA in 23.5% of patients with T1D and 29% of patients with T2D. The other triggering factors included pancreatitis, medication supply issues, and surgery **(**Fig. 1**)**. The biochemical evolution of DKA was similar among patients with T1D and T2D, with the only significant differences seen in venous lactate levels at 2, 4, and 6 h, which were higher in patients with T2D compared with patients with T1D **(**Fig. 2**).** Bicarbonate, pH, and blood glucose changes in the first 6 h of DKA admission were not different among the four groups. EDKA (defined as glucose less than 11.1 mmol/L at the time of DKA diagnosis, was diagnosed in 40 patients) [16]. Out of these 40 patients, 17 (42.5%) patients had PT1D, 17 (42.5%) had PT2D, four (10%) had NT1D, and two (5%) had NT2D. Among the 40 patients with EDKA, nine (22.5%) were on SGLT2i.

**Table 2:** Differences in biochemical results at admission among new and pre-existing type 1 and type 2 diabetes mellitus patients with index DKA admissions. Data presented as Median (IQR), Mean (± SD), and N (%) as appropriate

Laboratory Investigations (at admission)	T1D (480)		T2D (442)		Significance (*P* value)
	**PT1D** **(332)**	**NT1D** **(148)**	**PT2D** **(266)**	**NT2D** **(176)**	
**HbA1c %**	11.1 ± 2.5	12.9 ± 2.6	11.9 ± 2.9	12.5 ± 2.8	0.3
**White blood cell count** **10^3/uL**	13.2 ± 7.1	12.3 ± 6.7	14.9 ± 7.9	13.9 ± 6.8	0.06
**Hemoglobin at admission** **g/dl**	13.8 ± 2	14.9 ± 1.5	13.9 ± 2.3	14.8 ± 2.3	< 0.001
**Platelet count** **10^3/uL**	325.6 ± 100.5	312.1 ± 102.8	296.1 ± 116	299.3 ± 105.8	0.13
**Urea** **mmol/L**	6.6 ± 3.9	6.5 ± 6.2	8.7 ± 7.8	7.8 ± 6.4	< 0.001
**Creatinine clearance** **ml/min**	101.9 ± 0.6	103.1 ± 51.5	96.6 ± 96.8	97.4 ± 56.8	< 0.001
**Sodium** **mmol/L**	133.5 ± 4.2	135 ± 6.2	131.9 ± 5.7	132.1 ± 6.4	< 0.001
**Potassium** **mmol/L**	4.6 ± 0.8	4.2 ± 0.7	4.6 ± 0.8	4.4 ± 0.9	0.003
**BHB** **mmol/L**	5.9 ± 2.5	6.1 ± 2.1	6 ± 3	6 ± 2.7	< 0.001
**CRP** **mg/L**	35.5 ± 64.7	26.2 ± 56.5	71.3 ± 109.7	86.8 ± 136.5	< 0.001
**Serum HCO3** **mmol/L**	10.9 ± 4.02	10.4 ± 4.4	11.1 ± 4.6	11.5 ± 4.6	0.07

*Abbreviations*: *BHB* Beta-hydroxybutyrate, *CRP* C-reactive protein, *HCO3* Bicarbonate, *DKA* diabetic ketoacidosis; n, number of patients, *sd* standard deviation, *T1D* type 1 diabetes mellitus, *T2D* type 2 diabetes mellitus, *PT1D* pre-existing type 1 diabetes mellitus, *NT1D* new type 1 diabetes mellitus, *PT2D* pre-existing type 2 diabetes mellitus, *NT2D* new type 2 diabetes mellitus

### Clinical outcomes

DKA severity, LOS in the hospital, DKA duration, need for intensive care unit (ICU), and in-hospital mortality is tabulated **(**Table 3**)**. Severe DKA was prevalent in all groups except PT1D, which had most patients with moderate DKA (42.1%) (*P* value 0.01). LOS in hospitals ranged from 2.5 ± 3.5 days in patients with PT1D to 6.8 ± 11.3 days in patients with NT2D (*P* value < 0.001). Similarly, the DKA duration ranged from 18.9 ± 4.2 h in patients with PT1D to 26.6 ± 21.1 h in patients with NT2D (*P* value < 0.001). The ICU admission rates were 32% in patients with PT2D, and 31% in those with NT2D, whereas the ICU admissions in patients with NT1D and PT1D were 22.3% and 16.6%, respectively (*P* value < 0.001). Seven patients had in-hospital mortality (three in the NT1D group, two in the PT2D group, and two in the NT2D group) (*P* value 0.04).

**Table 3:** Clinical outcomes of patients with new and pre-existing type 1 and type 2 diabetes mellitus patients in the index DKA admissions. Data presented as Median (IQR), Mean (± SD), and N (%) as appropriate

Clinical Outcomes	T1D (480)		T2D (442)		Significance
	**PT1D** **(332)**	**NT1D** **(148)**	**PT2D** **(266)**	**NT2D** **(176)**	
**DKA severity**					0.01
Mild (202)	59 (17.8)	28 (18.9)	64 (24.1)	51 (29)	
Moderate (332)	140 (42.2)	49 (33.1)	88 (33.1)	55 (31.2)	
Severe (388)	133 (40.1)	71 (50)	114 (42.9)	70 (39.8)	
**LOS in the hospital (days)**	2.5 ± 3.5	4.3 ± 5.4	5.6 ± 9.1	6.8 ± 11.3	< 0.001
**DKA duration (hours)**	18.9 ± 4.2	23.1 ± 18.6	23.4 ± 18	26.6 ± 21.1	< 0.001
**In-hospital mortality**	0	3 (2)	2 (0.8)	2 (1.1)	0.04
**ICU admission**	55 (16.6)	33 (22.3)	86 (32.3)	55 (31.2)	< 0.001

*Abbreviations*: *DKA* diabetic ketoacidosis, *LOS* length of stay, *ICU* intensive care unit; n, number of patients, *sd* standard deviation, *T1D* type 1 diabetes mellitus, *T2D* type 2 diabetes mellitus, *PT1D* pre-existing type 1 diabetes mellitus, *NT1D* new type 1 diabetes mellitus, *PT2D* pre-existing type 2 diabetes mellitus, *NT2D* new type 2 diabetes mellitus

## Discussion

We have presented one of the largest regional data on a diverse patient population showing the comparison between the different clinical and biochemical characteristics in patients of newly diagnosed and pre-existent T1D and T2D with index DKA admissions. Our study cohort had an equal proportion of patients with T1D and T2D. Patients with new and pre-existent T1D and T2D had different demographics and clinical characteristics, which translated into different DKA outcomes. Patients with PT1D were predominantly females, whereas all other groups had a male preponderance. The ethnic-specific BMI showed that patients with PT1D and NT1D were mainly within the normal range. On the other hand, patients with PT2D and NT2D had a significant proportion of the overweight and obese population. Comorbid conditions such as hypertension, dyslipidemia, and microvascular and macrovascular DM complications were predominantly seen in patients with PT2D, followed by NT2D, PT1D, and, lastly, NT1D. The metabolic risk was highest in PT2D patients, whereas the lowest metabolic risk was identified in patients with NT1D. Although patients with PT2D had the worst health status (such as older age, more comorbidities, more micro and macrovascular DM complications, and the highest metabolic risk), LOS in the hospital and DKA duration were the longest in patients with NT2D (6.8 days and 26.6 h, respectively). ICU admissions were needed in 31.2% and 32.3% of patients with NT2D and PT2D, respectively. However, patients with T1D had better outcomes, with the shortest LOS in hospital and DKA duration in PT1D (2.5 days and 18.9 h, respectively) and the least frequency of ICU admissions. The evolution of DKA regarding serial laboratory investigations was similar in both DM types, except for serial lactate levels, which were higher in patients with T2D compared with those with T1D at 2, 4, and 6 h after admission. While a similar biochemical evolution reflects the same treatment of DKA in both DM types, outcomes do carry significant differences, mainly owing to different patient characteristics and DM type and duration.

We found an equal proportion of T1D and T2D among patients with DKA. This is different from other cohorts in which most patients had T1D. Ooi et al. reported 786 consecutive DKA episodes in a cohort with 75% of patients with T1D and 25% of patients with T2D [17]. Similarly, Xu et al. reported 64% and 36% of patients with T1D and T2D, respectively, in 531 patients with DKA hospitalizations [1]. In another cohort of 5,544 DKA-related hospitalisations, Ebrahimi et al. reported T2D in 30% of patients [18]. Among patients with newly diagnosed DM, there was also a similar proportion of patients with T1D and T2D, which is different from other cohorts. Newton et al. conducted a comparative study (*n* = 176) on new versus pre-existing T1D and T2D in patients with DKA in 2004. In their study, PT1D comprised the majority (*N* = 78, 65%) of DKA admissions, whereas NT1D and NT2D comprised 2.8% (5 patients) and 17% (30 patients) of total DKA admissions, respectively [12]. More recent data supports an increasing prevalence of DKA in patients with T2D. Lyerla et al. described DKA episodes in a population with predominantly T2D (56%) [19]. Similarly, Charoenpiriya et al. reported a higher T2D patient population (54%) compared with T1D (46%) in a recent 2-year prospective study on patients admitted with DKA [14]. The most elaborative results come from the study conducted by Zhong et al., which comprised more than 4000 DKA admissions from 1998 to 2013 [13], showing an annual increase of 4.2% in the incidence of DKA in patients with T2D. Our results consolidate these findings, which indicate that DKA trends have changed and shifted to a balanced proportion of T1D and T2D compared with a previously predominant patient population with T1D.

Many factors have played a role in the increasing occurrence of DKA in patients with T2D, such as prolonged exposure to hyperglycemia. Many patients with newly diagnosed T2D, in fact, have relatively long-standing undiagnosed DM, which is translated as the presence of micro and macrovascular complications in such patients. This is primarily due to poor access to healthcare and relatively lower screening rates in some parts of the world [20]. This prolonged undetected hyperglycaemic state poses risks of acute and chronic complications of DM, albeit a new diagnosis. More younger patients are being affected by T2D, and this population tends to have a rapid decline in beta-cell function, increasing the risk of DKA [21]. Some studies have shown insulin deficiency as a significant driver behind this development in the young population [22], and it may therefore be expected that the incidence of DKA in patients with T2D may soon surpass T1D if this trend persists. Although public health specialists do argue that screening of chronic diseases at younger ages can prevent their development and progression, the younger population are not routinely screened for chronic disorders [23]. Patients with PT2D generally have poor glycaemic control with consequent insulin deficiency, leading to DKA. The factors contributing to this poor glycaemic control in patients with T2D include comorbidities, lack of self-monitoring of blood glucose levels, metabolic risks, and other factors [24]. As the T2D population is generally older and sicker than the T1D population, this change in the trends of type and duration of DM among DKA admissions may have a significant impact on the clinically relevant outcomes of DKA.

Interestingly, patients with NT2D in this study had worse DKA-related outcomes compared to other groups. These patients had the longest LOS in the hospital and the longest duration while in DKA, more than those with PT2D. LOS and DKA duration was much less in T1D subsets. Data from several studies have identified an association between T2D with a higher LOS in hospitals, with some studies reporting a four times more prolonged LOS in patients with T2D compared with T1D [1, 17]. A higher LOS in the hospital among patients with new or old T2D can be multifactorial. First, these patients usually have one or more comorbidities, slowing the recovery of DKA compared to patients with T1D. Additionally, some of the comorbidities in patients with T2D, such as chronic kidney disease, ischemic heart disease, and non-alcoholic fatty liver disease, may limit or slow down glucose management. Second, patients with NT2D who present for the first time with DKA or patients with PT2D who are on oral medication and present with DKA and poor glycaemic control require the initiation of insulin before discharge. Hence, more time is required for insulin education and insulin titration. Lastly, T2D is associated with a lower socioeconomic status [25]. The relatively poor nutritional and educational status can translate into higher clinical needs and more diabetes education compared to patients with T1D, increasing the LOS in the hospital, DKA duration, and more adverse outcomes. A longer LOS and DKA duration in a patient with NT2D compared to PT2D could partly be due to the underlying prolonged unmanaged hyperglycaemic state, which leads to a higher oxidative stress in the body, hence leading to possibly poor clinical outcomes [26]. However, data regarding such differences is limited and needs more extensive evaluations.

Epidemiological data identifying demographic and clinical differences in patients with new and pre-existing DM in various DM complication cases, such as DKA, are needed from different ethnic populations. Additionally, the high prevalence of T2D affecting the younger population, in addition to poor screening methods in large parts of the world, is resulting in a poor global T2D-related health burden. This calls for urgent steps to ensure regular screening to detect DM at earlier stages which can be controlled and possibly reverted before acute complications such as DKA can happen. A comprehensive national DM screening program is already in place in Qatar with similar aims [27]. More effective and global policies are crucial in overcoming the increasing burden of poor DM-related outcomes.

The principal strength of our study lies in its extensive and diverse patient population, making the results generalisable to a vast geographic area. We identified and collected data from the index DKA admissions to find the most reliable proportion of DM types. The application of ADA DKA diagnostic criteria resulted in the exclusion of patients from the cohort but ensured an accurate representation of the cohort. Our study is, however, limited by its retrospective nature, due to which some covariates inherently could not be collected and might have influenced the results, such as anti-GAD Abs and C-peptide levels. This also limited our ability to validate the type of DM for each patient.

## Conclusion

T2D is increasingly causing DKA and causing a paradigm shift from a very low prevalence among the DKA population to an almost equal prevalence to T1D. DKA in T2D, especially NT2D, tends to have adverse outcomes. This can result in an increasing burden on healthcare and poor patient related DKA outcomes. As diabetes is influenced by environmental, geographical, and genetic variations, data presented in this study will help understand the differential evolution of DKA in similar patient populations. More studies from other ethnic groups are needed for a broader and global understanding of the epidemiological, clinical, and biochemical differences in DKA admissions based on the type and duration of DM.

## Data Availability

The datasets used and/or analysed in the current study are available from the corresponding author on reasonable request.
